# PD-1 deficiency is not sufficient to induce myeloid mobilization to the brain or alter the inflammatory profile during chronic neurodegeneration

**DOI:** 10.1016/j.bbi.2018.08.006

**Published:** 2018-10

**Authors:** J. Obst, R. Mancuso, E. Simon, D. Gomez-Nicola

**Affiliations:** Biological Sciences, University of Southampton, United Kingdom

**Keywords:** Programmed cell death-1, Myeloid cells, Neuroinflammation, Prion disease

## Abstract

Innate immune activation is a major driver of neurodegenerative disease and immune regulatory pathways could be potential targets for therapeutic intervention. Recently, Programmed cell death-1 (PD-1) immune checkpoint inhibition has been proposed to mount an IFN-γ-dependent systemic immune response, leading to the recruitment of peripheral myeloid cells to the brain and neuropathological and functional improvements in mice with Alzheimer’s disease-like β-amyloid pathology. Here we investigate the impact of PD-1 deficiency on murine prion disease (ME7 strain), a model of chronic neurodegeneration. Although PD-1 was found to be increased in the brain of prion mice, the absence of PD-1 did not cause myeloid cell infiltration into the brain or major changes in the inflammatory profile. However, we observed a slight exacerbation of the behavioural phenotype of ME7 mice upon PD-1 deficiency. These results do not support the possibility of using immune checkpoint blockade as a therapeutic strategy in neurodegenerative disease.

## Highlights

• The expression of PD-1 is increased during prion disease.
• Genetic deficiency of PD-1 does not affect myeloid cell trafficking into the brain.
• PD-1 deletion does not affect the inflammatory profile in prion disease.
Mice deficient in PD-1 showed a slightly accelerated progression of prion disease.

## 1 Introduction

Neuroinflammation is a central component of the pathophysiology of neurodegenerative disorders, such as Alzheimer’s disease (AD), Parkinson’s disease (PD), frontotemporal dementia (FTD), amyotrophic lateral sclerosis (ALS) or prion disease (Heneka et al., 2015a, Obst et al., 2017). It is characterised by a robust innate immune response with microglial proliferation and activation, and production of inflammatory cytokines and chemokines (Heneka et al., 2015). The importance of neuroinflammation as a component of neurodegenerative disorders has been highlighted by multiple genome-wide association studies identifying multiple loci associated with susceptibility for developing AD, many of them expressed in microglia (Jun et al., 2010, Guerreiro et al., 2013, Jonsson et al., 2013, Gjoneska et al., 2015, Sims et al., 2017, Huang et al., 2017). Whereas this implies a relevant role of innate immunity in the neurodegenerative process, recent studies have revealed that the adaptive immune response may also play a crucial role by limiting and shaping inflammation in the brain (Marsh et al., 2016).

Within this context, immune checkpoints are regulatory pathways that play a major role in maintaining the homeostasis of the immune system (Pardoll, 2012). The inhibition of these checkpoints has been previously exploited in the context of cancer immunotherapy in order to mobilise the immune system to target cancer cells (Pardoll, 2012, Lesokhin et al., 2015, Schachter et al., 2017). Some effort has been recently aimed to assess the potential effect of blocking the programmed cell death-1 (PD-1) pathway in mouse models of AD. However, conflicting data has been generated as to whether the inhibition of PD-1 by blocking antibodies has a disease modifying effect in AD mouse models (Baruch et al., 2016, Latta-Mahieu et al., 2017). On the one hand, Baruch et al. (2016) reported that PD-1 blockade in 5xFAD or APP/PS1 mice evokes an INFγ-dependent immune response which triggers peripheral monocytes infiltration into the CNS and enhances amyloid beta (Aβ) clearance. On the other hand, Latta-Mahieu et al. (2017) showed that immunotherapy against PD-1 by itself is not sufficient to reduce amyloid pathology in APP/PS1 mice. In this study, we aimed to clarify the role of PD-1 in neurodegenerative disease by analysing the impact of a genetic deletion of PD-1 in a model of prion disease, which combines misfolded protein accumulation and neurodegeneration (Obst et al., 2017), and where neuroinflammation plays a critical role (Gomez-Nicola et al., 2013). Our results showed that although PD-1 ablation led to an upregulation of CCL2 expression, this was not enough to modify the overall neuroinflammatory response or to induce a recruitment of monocytes to the prion-diseased brain, thereby having no major impact on the disease course of the mice.

## 2 Methods

### 2.1 Data mining from deposited RNA-seq and microarray datasets

To assess cellular expression of PD-1 (Pdcd1) and its ligands PD-L1 (CD274) and PD-L2 (PDCD1LG2), we used a brain cell type-specific RNAseq transcriptome database generated from purified neurons, astrocytes, oligodendrocyte precursor cells, newly formed oligodendrocytes, myelinating oligodendrocytes, microglia, endothelial cells and pericytes from mouse cerebral cortex (Zhang et al., 2014a). Datasets are publicly available on the website https://web.stanford.edu/group/barres_lab/brain_rnaseq.html.

To assess expression of PD-1 in the ME7 prion mouse brain compared to healthy brain, we mined expression data for selected genes from microarray datasets generated previously from brain homogenates (Lunnon et al., 2011). Datasets are publicly accessible at the NCBI gene Expression Omnibus (GEO) under GEO series accession code GSE23182 (http://www.ncbi.nlm.nih.gov/geo/ GSE23182).

### 2.2 Experimental model of prion disease

C57BL/6J mice (Harlan laboratories), termed WT throughout the manuscript, and Pdcd1^−/−^ mice (Nishimura et al., 1998), termed PD-1 KO throughout the manuscript, were bred and maintained in local facilities. Mice were housed in groups of 4–10, under a 12 h light/12 h dark cycle at 21 °C, with food and water *ad libitum*. To induce prion disease, 6 weeks old WT and PD-1 KO mice were anesthetized with a ketamine/xylazine mixture (85 and 13 mg/kg), and 1 μl of ME7-derived (ME7 animals) brain homogenate (10% w/v) was injected stereotaxically and bilaterally at the dorsal hippocampus, coordinates from bregma: anteroposterior, −2.0 mm; lateral, ±1.7 mm; depth, −1.6 mm. Naïve animals of both genotypes were used as control. All procedures were performed in accordance with U.K. Home Office licensing. Group sizes were defined after performing power calculations, in order to achieve a significant difference of p < 0.05, in light of a retrospective analysis of our previous published results, to reach a power between 0.80 and 0.90, depending on the specific experimental conditions. The effect size was calculated taking into consideration the strength of association between the variables, the sensitivity and the variation of any dependent variable. The calculations are the customary ones based on normal distributions.

### 2.3 Behavioural tests

Behavioural tests performed to detect the onset and progression of behavioral abnormalities in ME7 were open-field locomotor activity and burrowing activity (Gomez-Nicola et al., 2013).

#### 2.3.1 Locomotor activity

The open-field tests were performed using an activity monitor software (Med Associated). The mice were placed in individual cages of 27 × 27 for a period of 5 min and the total distance travelled was recorded. The average speed was used as an internal control of the mouse motor abilities.

#### 2.3.2 Burrowing

Plastic cylinders, 20 cm long and 6.8 cm in diameter, were filled with 190 g of normal diet food pellets and placed in individual mouse cages. Mice were placed individually in the cages for 2 h and overnight, and the remaining pellets were weighed at the end of each session and the amount displaced (“burrowed”) was calculated. The mice were then returned to their home cage. The data was expressed as weight of food displaced from the cylinder.

### 2.4 Fluorescent activated cell sorting (FACS) analysis

Mice were terminally anesthetized with an overdose of sodium pentobarbital and transcardially perfused with heparinised PBS. Brain hemispheres were harvested in PBS 2%FCS 2 mM EDTA (FACS buffer), mechanically triturated and enzymatically dissociated using the Neural Tissue Dissociation Kit (P) (Miltenyi). Then, samples were passed through a cell strainer of 70 μm mesh (BD2 Falcon) with FACS buffer, and centrifuged twice at 500*g* for 10 min at 4 °C. After the second wash, cells were resuspended in 37% Percoll (GE Healthcare) and centrifuged at 500*g* for 30 min at 18 °C (Grabert et al., 2016, Pino and Cardona, 2011). The supernatant and myelin layers were discarded, and the cell pellet enriched with microglia/macrophages was resuspended in FACS buffer. Samples were split in several tubes and immunostained. Primary antibody labeling was performed for 1 h at 4 °C, using the following primary antibodies: rat-anti-mouse CD11b (BD Biosciences, clone M1/70), rat-anti-mouse CD45 (Biolegend, clone 30-F11), rat-anti-mouse CD3 (Biolegend, clone 17A2) and rat-anti-mouse Ly6C (BD Biosciences, clone AL-21), adding 7-Aminoactinomycin D (7-AAD, Biolegend) as a cell viability marker. Moreover, unstained cells and isotype-matched control samples were used to control for autofluorescence and/or non-specific binding of antibodies. In addition, blood samples were harvested by cardiac puncture and collected in EDTA tubes. Spleens were minced and passed through 70 um cell strainers. Leukocytes in blood and spleen were stained with rat-anti-mouse CD11b (BD Biosciences, clone M1/70), rat-anti-mouse CD45 (Biolegend, clone 30-F11), rat-anti-mouse CD3 (Biolegend, clone 17A2) and rat-anti-mouse Ly6C (BD Biosciences, clone AL-21) and Fixable Viability Dye eFluor™ 450 (eBioscience), using the same method described above. Erythrocytes were then lysed in RBC lysis buffer (eBioscience). Samples were run on a BD FACS Aria Flow cytometer. Data was analysed using FlowJo software.

### 2.5 Analysis of gene expression by qPCR

Brain hemispheres were homogenized in Trizol reagent (Invitrogen), following the manufacturer instructions to isolate RNA and as previously described (Gomez-Nicola et al., 2013). The isolated RNA was quantified (Nanodrop ND-1000, Thermo Scientific) and 1 μg of total RNA retrotranscribed with iScriptTM cDNA Synthesis Kit (Biorad). cDNA libraries were analyzed by RT-PCR using iTaqTM Universal SYBRVR Green Supermix (Biorad) for the following genes (Sigma-Aldrich): Ccl2 (NM_011333.3), forward (FW), TTAAAAACCTGGATCGGAACCAA; reverse (RV), GCATTAGCTTCAGATTTACGGGT; Csf1 (NM_007778.4), FW, AGTATTGCCAAGGAGGTGTCAG; RV, ATCTGGCATG-AAGTCTCCATTT; Csf1r (NM_001037859.2), FW, GCAGTACCACCATCCACTTGTA; RV, GTGAGACACTGTCCTTCAGTGC; Il-10 (NM_010548.2), FW, GGCCCAGAA-ATCAAGGAGCA; RV, ACAGGGGAGAAATCGATGACAG; Il-1β (NM_008361.4), FW, CAAAAGATGAAGGGCTGCTTCC; RV, ATGTGCTGCTGCGAGATTTG; Il-34 (NM_001135100.2), FW, CTTTGGGAAACGAGAATTTGGAGA; RV, GCAATCCTGT-AGTTGATGGGGAAG; Il-4 (NM_021283.2), FW, AGCAACGAAGAACACCACAG; RV, GCATCGAAAAGCCCGAAAGAG; Il-6 (NM_001314054.1), FW, CTCTGCAAGA-GACTTCCATCC; RV, TGAAGTCTCCTCTCCGGACT; Ifn-γ (NM_008337.4), FW, ACAATGAACGCTACACACTGCAT; RV, TGGCAGTAACAGCCAGAAACA; Ppia (NM_008907.1), FW, AGGGTGGTGACTTTACACGC; RV, CTTGCCATCCAGCCATTCAG; Tgf-β (NM_011577.2), FW, TGTACGGCAGTGGCTGAACC; RV, CGTTTGGG-GCTGATCCCGTT; Tnf-α (NM_001278601.1), FW, GGCAGGTTCTGTCCCTTTCAC; RV, TTCTGTGCTCATGGTGTCTTTTCT; Pdcd1 (NM_008798.2), FW, TCTACCTC-TGTGGGGCCATC; RV, CCTCCTTCAGAGTGTCGTCCT.

The integrity of the PCR products was checked in a 1.2% agarose gel. Threshold cycle (Ct) values were calculated using delta delta Ct method, using Peptidylprolyl isomerase A (Ppia) as housekeeping gene.

### 2.6 Cresyl violet staining

Coronal hippocampal sections of 30 μm thickness were cut on a cryostat, mounted on glass slides, dehydrated and incubated in 100% xylene for 15 min to de-fat the tissue. Afterwards, tissue sections were rehydrated and incubated in an acidified solution of 3.1 mM cresyl violet for 10 min. Sections were washed in tap water, dehydrated and mounted with DPX. Neurodegeneration in the hippocampus was quantified measuring the thickness of the CA1 pyramidal layer, by sampling and averaging at least 7 values across the CA1 region per section (5–8 sections/mouse; n = 3–4 mice/group).

### 2.7 Statistics

Data are shown as mean ± SEM and where analysed using the GraphPad Prism 6 software package (GraphPad Software). Data were analysed using two-way ANOVA with Tukey post-hoc test for multiple comparisons, Student’s *t*-test or one-way ANOVA followed by a Tukey post-hoc test for multiple comparisons, as indicated. Differences were considered significant at p < 0.05.

### 2.8 Study approval

All procedures were performed in accordance with U.K. Home Office licensing and following guidance from institutional review committees.

## 3 Results

### 3.1 PD-1 expression is increased in the ME7 model of prion disease

**Fig. 1:**
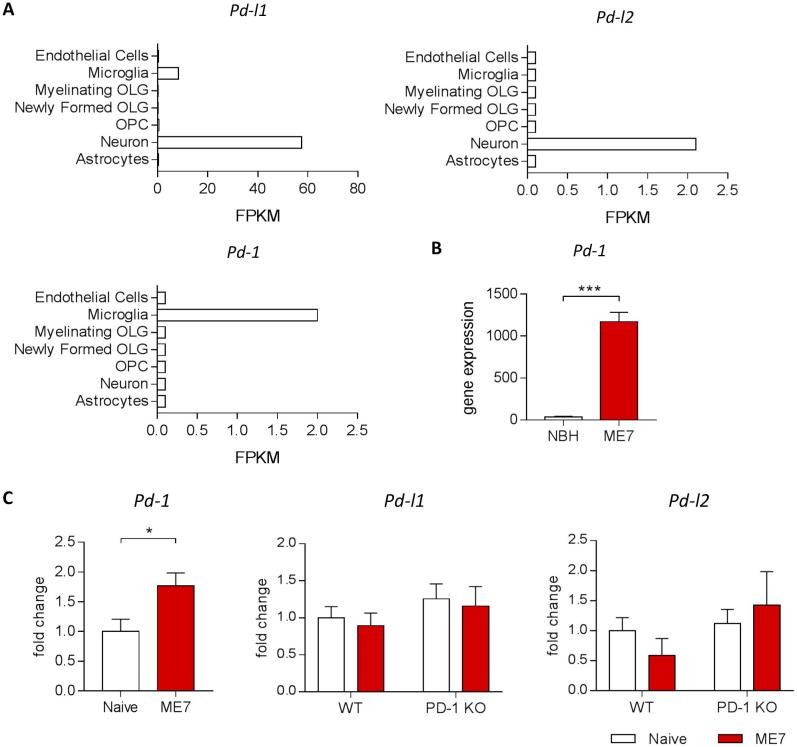
PD-1 expression in the ME7 model of prion disease. (A) RNAseq analysis of PD-L1, PD-L2 and PD-1 show cell-specific expression of PD-1 by microglia and the ligands by neurons in the healthy brain. (B) Microarray expression intensities in whole brain homogenates demonstrate increased expression of PD-1 in ME7 compared to control mice. (C) qPCR analysis demonstrated increased expression of PD-1, while PD-L1 and PD-l2 remain unchanged in the brain of ME7 prion mice at 18 weeks post-induction compared to naïve control mice. Data shown represent Mean ± SEM, n = 8 per group. Student’s *t*-test, ^*^p < 0.05, ^***^p < 0.001. RNAseq data were obtained from publicly accessible datasets on the website https://web.stanford.edu/group/barres_lab/brain_rnaseq.html (Zhang et al., 2014a). Microarray data were re-analyzed from publicly available datasets (GSE23182) (Lunnon et al., 2011), with log FC: 5.09; p-value: 0.00002714; adjusted p-value: 0.01301.

To gain an initial understanding of the expression pattern of PD-1 and its ligands in the brain, we first made use of a brain cell type-specific RNAseq transcriptome database publicly accessible on the website https://web.stanford.edu/group/barres_lab/brain_rnaseq.html (Zhang et al., 2014b). By obtaining cell-specific expression data from this database we observed expression of the ligands PD-L1 and PD-L2 mainly by neurons, while PD-1 expression was found to be restricted to microglia in the healthy brain (Fig. 1A). Then, we re-analyzed microarray data from publicly accessible datasets (GSE23182) (Lunnon et al., 2011), which showed that in the context of prion disease pathology PD-1 expression was highly increased compared to controls (Fig. 1B). As determined by qPCR, PD-1 deficiency did not cause changes in gene expression of the two ligands in the brain (Fig. 1C). While we did not observe differences in gene expression of the ligands between naïve and ME7 prion mice, PD-1 expression was significantly increased in the brain of mice infected with prion disease, confirming the results of the microarray (Fig. 1B, C).

### 3.2 Genetic deletion of PD-1 does not alter myeloid cell populations in the healthy brain and in the systemic compartment, but increases lymphocyte recruitment to the brain

**Fig. 2:**
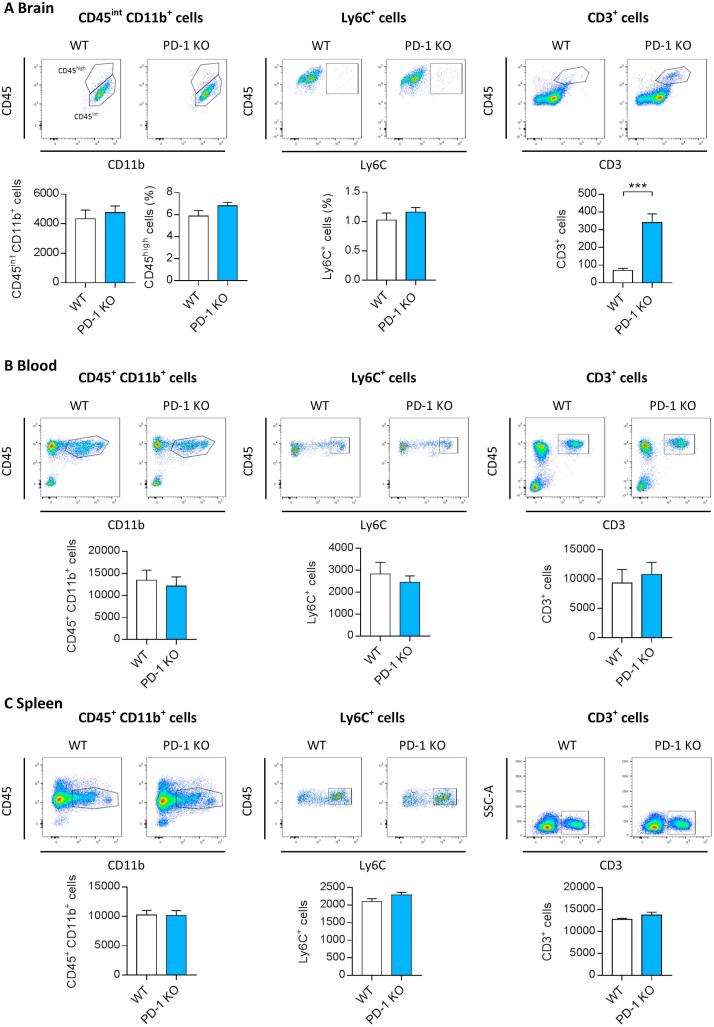
Characterization of myeloid/lymphocyte cell populations in mice deficient in PD-1. Flow cytometric analysis of myeloid and lymphocyte populations in brain, blood and spleen of WT and PD-1 KO mice at the age of 18 weeks. (A) Representative dot plots and quantification of the number of brain microglia, defined as CD11b+ CD45int, and brain macrophages, defined as CD11b+ CD45high or Ly6C+ show no changes upon genetic deletion of PD-1. Number of CD3+ T cells is increased in PD-1 KO mice compared to WT controls. Monocytes/macrophages (CD11b+ CD45+) and T cell (CD3+) frequencies in the blood (B) and spleen (C) are not affected by the absence of PD-1. Analysed populations are depicted in encircled gating. Data shown represent Mean ± SEM, n = 8 per group. Student’s *t*-test, ^***^p < 0.001.

We next characterized the frequency of myeloid and lymphoid cell populations in the brain and in peripheral compartments (blood and spleen) in healthy PD-1 KO mice compared to WT controls, to determine any baseline differences inherent due to PD-1 deficiency. In the brain, FACS analysis revealed no differences between WT and PD-1 KO mice in microglial cells (CD45^int^ CD11b^+^) and infiltrating monocytes (CD45^high^ CD11b^+^ Ly6C^+^) (Fig. 2A), indicating that there is no impact of the absence of PD-1 on myeloid cell populations in the healthy mouse brain. The number of T cells in the brain, defined as CD45^+^ CD3^+^ cells, was significantly increased in mice deficient in PD-1 compared to WT mice, indicating an increased infiltration of these peripheral immune cells in the absence of PD-1 (Fig. 2A). This increased T cell frequency in the brain was not accompanied by increased numbers of T cells in the systemic compartment, since we did not observe higher numbers of T cells in the spleen or blood of PD-1 KO mice (Fig. 2B, C). However, we cannot exclude that the observed changes are the result of a potential genetic drift that occurred between the WT and PD-1 KO, since animals were not littermates. FACS analysis of CD45^+^ CD11b^+^ blood monocytes and spleen macrophages, as well as classical Ly6C^+^ monocytes/macrophages in blood and spleen, did not show any differences in terms of number in mice lacking PD-1 compared to WT (Fig. 2B, C). In summary, our data suggests that PD-1 deficiency leads to increased trafficking of T cells to the brain, without affecting the number of microglia or monocyte/macrophage populations in the systemic compartment.

### 3.3 PD-1 deficiency does not affect microglial numbers or the infiltration of peripheral myeloid cells into the brain of ME7-prion mice

**Fig. 3:**
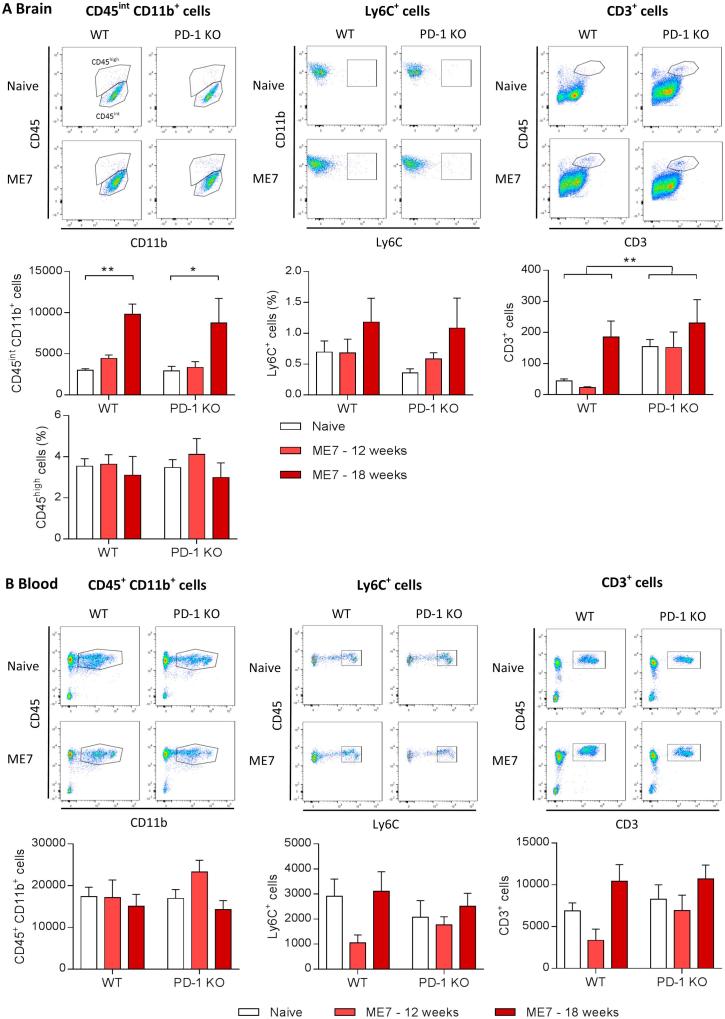
Characterization of myeloid/lymphocyte cell populations in ME7 prion mice deficient in PD-1. Flow cytometric analysis of myeloid and lymphocyte populations in brain and blood of naïve or ME7-infected WT and PD-1 KO mice at 12 and 18 weeks post-induction (wpi). (A) Representative dot plots and quantification of the number of brain microglia (CD11b+ CD45int), brain macrophages (CD11b+ CD45high or Ly6C+) and brain CD3+ T cells show no differences upon genetic deletion of PD-1. (B) Monocytes/macrophages (CD11b+ CD45+) and T cell (CD3+) frequencies in the blood of naïve and ME7 prion mice demonstrate no changes in the absence of PD-1. ME7 dot plots depict 18 wpi. n = 4–8 per group for blood analysis, n = 3–11 for brain analysis. Data shown represent Mean ± SEM, Two-way ANOVA followed by Tukey’s multiple comparison test. ^*^p < 0.05, ^**^p < 0.01.

We next wanted to determine if the absence of PD-1 affects the number of microglia in the brain, as the main brain-resident cells expressing it (Fig. 1A), as well as the trafficking of monocytes from the blood to the brain, in mice infected with the prion strain ME7. In agreement with previous findings (Gomez-Nicola et al., 2013), ME7 prion mice show an age-dependent increase in CD45^int^ CD11b^+^ microglia cells in the brain, as determined by FACS analysis, which was not changed by a deficiency of PD-1 (Fig. 3A). Furthermore, there was no difference in the percentage of CD45^high^ and Ly6C^+^ cells in prion mice deficient in PD-1, indicating that there was no effect on recruitment or infiltration of peripheral myeloid cells into the brain during prion disease in the absence of PD-1 (Fig. 3A). Numbers of CD3^+^ T cells are significantly higher in the brain of PD-1 KO mice compared to WT control, and there was a trend towards increased numbers during the course of prion disease in WT mice, which did not further increase in the absence of PD-1 (Fig. 3A), demonstrating there is not an increased influx of T cells into the prion brain upon PD-1 deficiency. We did not observe an effect on circulating monocyte and T cell frequencies in the blood in prion-infected mice, and the absence of PD-1 did not cause any significant alterations in the immune cell composition compared to control (Fig. 3B). Taken together, these data do not hint to the possibility that ablation of immune checkpoint molecule PD-1 affects the microglia population or recruitment of peripheral myeloid cells into the brain during prion protein pathology.

### 3.4 The inflammatory profile in the brain of ME7 prion mice deficient in PD-1 remains largely unaltered

**Fig. 4:**
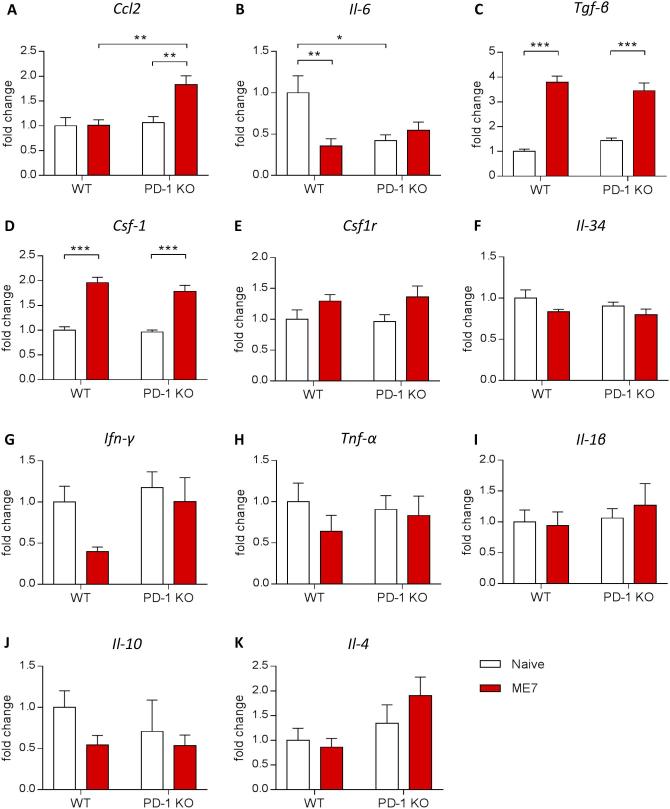
Brain inflammatory profile in ME7 prion mice deficient in PD-1. Quantitative PCR analysis of inflammatory markers in whole brain homogenates of naïve or ME7-infected WT and PD-1 KO mice at 18 weeks post-induction. PD-1 KO mice show decreased expression of Il-6 at baseline that is unchanged in the context of disease, while Ccl2 expression is increased in prion mice in the absence of PD-1 compared to WT control. n = 7–10 mice per group. Data shown represent Mean ± SEM, Two-way ANOVA followed by Tukey’s multiple comparison test. ^*^p < 0.05, ^**^p < 0.01, ^***^p < 0.001.

As the immune response is a key component of the progression of neurodegenerative diseases, we investigated if the disruption of the PD-1 gene could have an impact on the inflammatory profile in brains from prion mice (Fig. 4). The induction of prion disease led to the previously described modifications in the expression of mediators of the immune response (Gomez-Nicola et al., 2013, Boche et al., 2006, Cunningham et al., 2005). In WT ME7 mice, the expression of the pro-inflammatory cytokine Il-6 was significantly downregulated while the expression of Tgf-β was significantly up-regulated compared to the WT naïve mice (Fig. 4B, C). In PD-1 KO ME7 mice the expression of the chemokine Ccl2 and Tgfβ were significantly up-regulated compared to naïve PD-1 KO mice (Fig. 4A, C). The expression of Il-6 was downregulated in PD-1 KO naïve mice compared to WT naïve mice (Fig. 4B). Interestingly, the disruption of PD-1 led to increased expression of Ccl2 in PD-1 KO ME7 mice compared to WT ME7 mice, correlating to previously reported effects of PD-1 inhibition (Fig. 4A) (Baruch et al., 2016). However, we did not observe any significant changes in IFNγ expression in the absence of PD-1, as has been previously described when PD-1 was pharmacologically blocked (Baruch et al., 2016). Regarding the expression of the other pro- or anti- inflammatory cytokines, we did not find any significant modifications of their expression with genotype or pathology.

The proliferation of microglia, one of the hallmarks of neurodegenerative diseases, is triggered by an increase in the expression of components of the CSF1R pro-mitogenic pathway (Gomez-Nicola et al., 2013, Olmos-Alonso, 2015), so here we investigated if the disruption in PD-1 could have an impact on the expression of Csf1r and its ligands Csf1 and Il-34 in the brain. As previously shown by our lab, we confirmed that the expression of Csf-1 was increased in WT ME7 mice compared to WT naïve mice (Fig. 4D) (Gomez-Nicola et al., 2013). However, the observed change was not modified by PD-1 KO. Regarding the expression of Csf1r and Il-34, we did not find any significant modifications of their expression with genotype or pathology (Fig. 4E, F).

### 3.5 PD-1 deficiency causes a mild exacerbation of the behavioral deficits, but does not affect neurodegeneration in prion disease mice

**Fig. 5:**
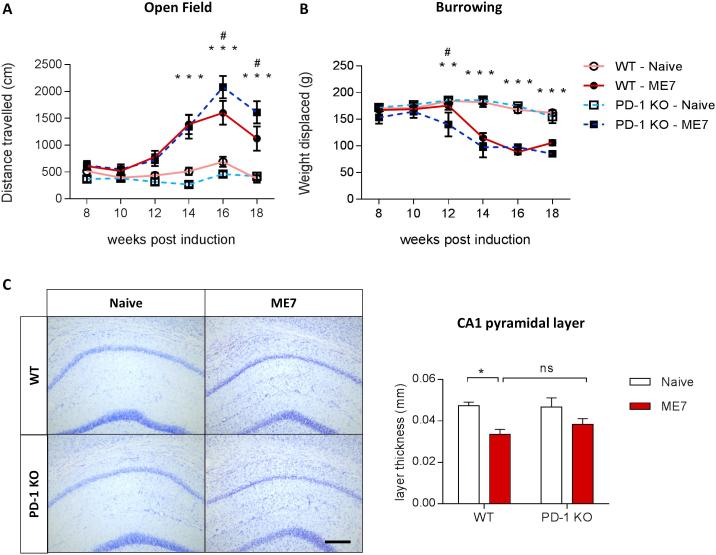
Impact of genetic deletion of PD-1 on cognitive deficits and hippocampal neurodegeneration in ME7 prion mice. (A) Locomotor activity determined by the distance travelled in the Open Field is increased in ME7 prion mice lacking PD-1 compared to WT controls, indicating elevated hyperactivity. (B) Deficits in burrowing activity are accelerated in ME7 mice in the absence of PD-1 compared to WT ME7 mice. n = 8–10 per group. (C) Cresyl violet staining of CA1 pyramidal neurons. n = 3–4 per group. Scale bar 100 μm. Data shown represent Mean ± SEM, Two-way ANOVA followed by Tukey’s multiple comparison test. ns – not significant, ^*^p < 0.05, ^**^p < 0.01, ^***^p < 0.001 wt – naïve compared to PD-1 KO – naïve and WT - ME7 compared to PD-1 KO – ME7, ^#^p < 0.05 PD-1 KO – ME7 compared to WT – ME7.

Consistent with previous reports (Gomez-Nicola et al., 2013), ME7 prion mice show a hyperactive phenotype, evidenced by increased distance travelled in the open field, which is further exacerbated by approximately 30% in ME7 mice deficient in PD-1 (Fig. 5A). ME7 mice also display reduced burrowing behavior at early stages, a sign of anhedonia typically described in prion-diseased mice. PD-1 ablation resulted in an earlier onset of anhedonia, from 14 to 12 weeks post induction, in WT and PD-1KO mice induced with prion disease, respectively (Fig. 5B). The degeneration of hippocampal pyramidal neurons in ME7 prion mice is evidenced by a thinning of the CA1 region, which is not significantly different from PD-1 KO ME7 mice (Fig. 5C). Taken together, behavioral analysis demonstrated a premature deterioration of burrowing and locomotor behavior in PD-1 KO mice infected with prion, indicating a slight exacerbation of disease phenotype in the absence of PD-1 immune checkpoint control, which is not accompanied by changes in neurodegeneration of the pyramidal neurons in the hippocampus.

## 4 Discussion

PD-1 is an immune-regulatory pathway known to suppress T-cell-mediated immune response and has been previously suggested to contribute to neuroinflammatory processes in neurodegenerative disease. In the light of recent reports describing conflicting results as to their effect on AD-related pathology (Baruch et al., 2016, Latta-Mahieu et al., 2017), we aimed to observe PD-1-mediated immunoregulatory effects in another model of neurodegenerative disease, the prion ME7 mouse model. Data taken from a microarray analysis showed that PD-1 expression is highly increased in ME7 mice (Lunnon et al., 2011) and we likewise observed an upregulation of PD-1 in the brain of prion mice, suggesting a possible role of this pathway in prion disease pathology. In the absence of PD-1 signaling we observed a slight exacerbation of the behavioral outcome, while myeloid cell frequencies, hippocampal neurodegeneration and the overall inflammatory profile in the brain were not changed, in spite of an overexpression of Ccl2.

It has been previously suggested that PD-1 immune checkpoint inhibition increases infiltration of peripheral myeloid cells into the brain, which is accompanied by an IFNγ-mediated immune response and increased Ccl2 mRNA levels particularly in the choroid plexus of the 5xFAD model of β-amyloidosis (Baruch et al., 2016). In our model of prion disease, we found a significant increase in the levels of Ccl2 transcript in the absence of PD-1, a chemokine which is a well-known inductor of the recruitment of myeloid cells into the CNS (Mildner et al., 2007). However, this increase in Ccl2 was not sufficient to either modify the number of microglia cells in the brain, or the infiltration of blood derived myeloid cells. The observation that there is no increased influx of peripheral myeloid cells into the brain is in agreement with reports which did not observe an effect on recruitment of peripheral immune cells in a model of EAE upon PD-1 deficiency (Zhang and Braun, 2014)and in a model of β-amyloidosis after systemic PD-1 blockade (Latta-Mahieu et al., 2017). Although the myeloid cell populations in the brain and the systemic compartment are unchanged upon PD-1 deficiency, we found increased numbers of T cells in the brain of PD-1 KO mice compared to WT, regardless of disease, which was not accompanied by increased T cell frequency in the blood. This indicates differentially regulated T cell trafficking into the brain in mice deficient in PD-1. Although we repeatedly observed increased T cell numbers in the brain in the absence of PD-1 in several independent experiments, this phenomenon has not yet been investigated in the context of PD-1 antibody administration. It is possible that increased T cell abundance in the brain is due to an effect of the constitutive deficiency of PD-1 throughout lifetime which is not apparent upon transient PD-1 blockade, however it has been previously described that anti-PD-1 treatment causes an increase in CD4^+^ (Baruch et al., 2016) or both CD4^+^ and CD8^+^ (Latta-Mahieu et al., 2017) T cell frequencies in the spleen. Thus, it would be of interest to also explore the dynamics of T cells in the brain following PD-1 antibody blockade. Since the characterization of the type of T cells accumulating in the brain in the absence of PD-1 was beyond the scope of this article, the functional consequences of this increased abundancy of T cells remains to be determined. Although the ME7 model of prion disease is not dominated by T cell responses, there is a trend towards increased numbers of T cells in WT mice, but no further increase of T cell frequencies during the course of pathology in PD-1 KO mice was observed, indicating that in terms of prion pathology T cell responses do not seem to contribute to disease phenotype in the absence of PD-1.

The inflammatory profile of the brain was largely unchanged upon PD-1 deficiency, except the observed changes in Ccl2 and Il-6 expression. While a previous report described an increase in IFNγ expression in response to immune checkpoint blockade in 5xFAD mice (Baruch et al., 2016), we did not observe a significant effect in the absence of PD-1 signaling in prion disease, although we did not determine IFNγ levels specifically in the choroid plexus but in whole brain preparations. While TGFβ signaling and components of CSF1R pathway were increased in ME7 prion mice, a phenotype that has been previously described (Gomez-Nicola et al., 2013, Boche et al., 2006, Cunningham et al., 2002), pro- and anti-inflammatory genes were generally not altered in ME7 mice deficient in PD-1, indicating that PD-1 does not have an significant impact on the inflammatory response in prion disease. However, in other models of neurodegeneration and injury, mixed effects have been described when PD-1 is lacking, ranging from the induction of an “M1”-like phenotype in spinal cord injury in PD-1 KO mice (Yao et al., 2014), to unchanged inflammatory markers in acute and chronic pain models after PD-1 blockade (Chen et al., 2017), to reduced inflammation in ischemic stroke upon anti-PD-L1 antibody treatment (Bodhankar et al., 2015), indicating that the effect of immune checkpoint blockade is diverse depending on the exact disease context and experimental conditions.

We investigated prion-induced neurodegeneration as a major pathological hallmark, observing that PD-1 deficiency did not alter the loss of pyramidal neurons in the hippocampus of ME7 mice, indicating that the absence of PD-1 did not affect prion pathology. However, we observed behavioral effects in PD-1-deficicent prion mice, implicating that there is a mild impact of PD-1 deficiency on disease progression. PD-1 KO mice showed a slight exacerbation in two different behavioral tasks when compared to WT mice infected with ME7, which points to the possibility that PD-1 ablation is more detrimental than beneficial in the context of neurodegeneration. An exacerbation of clinical symptoms in PD-1 KO mice has been observed before in models of EAE (Zhang and Braun, 2014) and intracerebral hemorrhage (Yuan et al., 2016). Although we did not proceed to identify the cellular or molecular basis underlying the acceleration of prion disease pathology, it is possible that PD-1 plays a role in multiple aspects of the neuroinflammatory response or that changes are very subtle and not detectable with the here applied methods. The investigation of the functional status of specific immune cell types could provide useful information concerning the mechanisms underpinning the behavioral impact. It is also conceivable that PD-1 could exert non-immune modulatory effects on specific neuronal populations, as it has been proposed before on sensory neurons in dorsal root ganglia in models of acute and chronic pain, where likewise inflammatory response was not affected by the absence of PD-1 (Chen et al., 2017). Further investigation is needed to elucidate by what mechanism PD-1 exerts its effect in the context of prion disease pathology. While previously antibody-mediated PD-1 immune checkpoint blockade, which is currently used as immunotherapy to treat cancer (Pardoll et al., 2012), has been suggested as a novel therapeutic strategy to tackle AD and potentially other neurodegenerative diseases (Baruch et al., 2016), a very recent publication showed that PD-1 blockade has no effect on Aβ pathology in 3 different models of AD (Latta-Mahieu et al., 2017). Although it has to be noted that our experiments follow a different strategy, using a genetic knockout mouse that is not directly comparable with the approach of an antibody administration, our data rather fit with the latter observations, since we did not find any evidence that targeting PD-1 is in any way effective to change the trajectory of the neuroinflammatory response in chronic neurodegeneration. On the contrary, it might more likely be detrimental in terms of clinical outcome, an aspect that would discourage its consideration as potential therapy for chronic neurodegenerative diseases including Alzheimer’s.

## 5 Author contributions:

J.O., R.M., E.S. and D.G.-N., designed the study, R.M. and J.O. performed flow cytometric analyses; E.S. performed qPCR analyses; J.O., E.S. and R.M. performed behavioral analysis; J.O. wrote the manuscript and R.M., E.S. and D.G.-N. contributed to drafting the final manuscript. All authors read and approved the manuscript.

## 6 Conflict of interest

The authors have declared that no conflict of interest exists.
